# *Clostridioides difficile* Infections and Antibiotherapy: Results of Four Years of Observation in a Romanian Tertiary Hospital

**DOI:** 10.3390/microorganisms12122490

**Published:** 2024-12-03

**Authors:** Carmen-Cristina Vasile, Luisa-Andreea Gheorghe, Carmen-Daniela Chivu, Marta Ana Maria Anghel, Ștefan Eduard Mîinea, Daniela Pițigoi, Maria-Dorina Crăciun

**Affiliations:** 1 Department of Epidemiology, Carol Davila University of Medicine and Pharmacy, 050474 Bucharest, Romania; carmen-cristina.vasile@rez.umfcd.ro (C.-C.V.); luisa-andreea.ilie@rez.umfcd.ro (L.-A.G.); marta-ana-maria.anghel@rez.umfcd.ro (M.A.M.A.); stefan-eduard.miinea@rez.umfcd.ro (Ș.E.M.); maria.craciun@umfcd.ro (M.-D.C.); 2 National Institute for Infectious Diseases “Prof. Dr. Matei Balș”, 021105 Bucharest, Romania; 3 National Administration of Penitentiaries Bucharest, 023762 Bucharest, Romania; 4 Emergency Clinical Hospital for Children “Grigore Alexandrescu”, 011743 Bucharest, Romania; 5 Medical Directorate, Ministry of Internal Affairs, 010919 Bucharest, Romania

**Keywords:** *Clostridioides difficile*, surveillance, incidence, antibiotherapy, tertiary hospital

## Abstract

*Clostridioides difficile* infection (CDI) is one of the main causes of morbidity associated with antibiotic use, producing both healthcare-associated infections and community infections. This study aims to describe the epidemiological characteristics, the clinical outcomes, previous antibiotic exposure, and other risk factors of hospitalized patients with CDI in a tertiary infectious disease hospital in Bucharest, Romania. We performed a descriptive analysis based on four-year surveillance data, collected in a tertiary infectious disease hospital in Bucharest, Romania. The annual incidence of CDIs varied from 65.1 cases per 10,000 discharges in 2020 to 211.7 cases per 10,000 discharges in 2023, with a continuously ascending trend. Most of the cases were hospital-acquired cases. There was a high share of antibiotic consumption three months before admission (61.3%). Third-generation cephalosporins, β-lactams with inhibitor combination, and carbapenems were the most used antibiotics, with shares of 46.0%, 25.2%, and 18.6%, respectively. Hospitalization in the previous 12 months and contact with a confirmed CDI case were other frequent factors in the study group, the occurrences of which were recorded as 66.2% and 2.4%, respectively. The surveillance data identified that the annual trend in CDIs is very variable, suggesting the need for continuous and multiannual analysis.

## 1. Introduction

*Clostridioides difficile (CD)* is an anaerobic, Gram-positive pathogen, one of the main causes of morbidity associated with antibiotic use, producing both healthcare-associated infections (HAIs) and community infections (CIs) [1,2]. The clinical spectrum ranges from asymptomatic cases, when the pathogen only colonizes the human large bowel, to symptomatic clinical outcomes, characterized by diarrhea, fever, abdominal pain, or life-threatening conditions due to toxic megacolon complications. These pathological pathways are explained by the main virulence factors of this bacteria, represented by enterotoxin A (TcdA) and cytotoxin B (TcdB) [3]. The clinical outcome and the recurrence of CD infection (CDI) depend on the virulence of the strains and the individual risk level of the patient [4,5].

Since 2004, new ribotypes (RTs) of CD have emerged worldwide and have been associated with increased morbidity and mortality [6,7,8,9,10,11,12]. The most frequent RTs in the European annual report for CDI during 2018 and 2020 were RT014/020, RT002, and RT078 [13].

The main risk factors associated with CDI and reported in published studies or guidelines are recent antibiotic exposure, gastrointestinal surgery or manipulation, a long length of stay in healthcare settings, underlying illness, immunocompromising conditions, and advanced age [13,14,15,16,17].

As a large epidemiological problem for hospitalized patients across the world, CDI is associated with high values of incidence and high mortality rates and is a critical burden on healthcare systems, as the management of hospitalized CD cases includes diagnostic, therapeutic, and prevention approaches [18].

The surveillance data showed that a large proportion of CD cases are hospital-acquired infections. Until 2016, no standardized surveillance systems existed in the European area. The European Centre for Disease Control and Prevention (ECDC) initiated a CDI surveillance system in 2016 [19]. Up to October 2024, two reports have been published regarding CDI in European countries. The incidence density of CDI between 2016 and 2017 was 3.48 cases per 10,000 patient days, with higher values registered in tertiary hospitals, and 1 of 26 patients with HAI-CDI deceased [20]. In 2020, the CDI surveillance data had insufficient coverage, due to the COVID-19 public health emergency. The annual reported incidence of HAI-CDI varied from 2.79 cases per 10,000 patient days in 2018 to 2.58 cases per 10,000 patient days in 2020. The incidence density of CI-CDI varied from 0.69 cases per 1000 admitted patients in 2018 to 1.35 cases per 1000 admitted patients in 2020 [13].

The National Center for Surveillance and Control of Communicable Diseases (NCSCCD), Romania, implemented the first national surveillance system for CDI in 2014, targeting all patients admitted to Romania’s public or private healthcare facilities [21]. Currently, CDI data are collected through a national surveillance system adjusted to European protocol and through the Point Prevalence Survey of HAIs and Antimicrobial Use (PPS) [19,21]. The NCSCCD surveillance reports noted a rise in CDI incidence after the start of the COVID-19 pandemic, with 48 cases per 10,000 discharged patients observed in 2020, 43 cases per 10,000 discharged patients observed in 2021, and 42 cases per 10,000 discharged patients observed in 2022 [22]. The Point Prevalence Survey of HAIs and Antimicrobial Use (PPS) in Romanian hospitals during 2023 reported that the most frequent HAI was CDI [22].

Antibiotherapy as a risk factor for CDI is an important topic for surveillance. Studies performed in infectious disease hospitals, which register a higher frequency of CDI cases and antibiotic use, highlight critical aspects of public health interventions.

There is an important difference regarding antibiotic use in the EU. The surveillance data and clinical studies classify Romania as a country with high antibiotic consumption, with values of 27.6 defined daily doses (DDD) per 1000 inhabitants per day in all sectors overall and 1.39 DDD per 1000 inhabitants per day in the hospital sector. The values registered at the European level were 19.4 DDD per 1000 inhabitants per day in all sectors and 1.61 DDD per 1000 inhabitants per day in the hospital sector [22,23,24]. The National Report regarding antibiotic consumption in 2020 and 2021 communicated that third-generation cephalosporins and fluoroquinolones were the primary classes of antibiotics most frequently administrated three months before CDI diagnosis [25].

CD remains an important pathogen that threatens individuals and populations. The available data regarding clinical symptomatic cases suggest the need for the continuous study of this public health issue, with variable trends regarding the most important indicators.

This study aims to describe the epidemiological characteristics, the clinical outcomes, previous antibiotic exposure, and other risk factors of hospitalized patients with CDI in a tertiary infectious disease hospital in Bucharest, Romania.

## 2. Materials and Methods

### 2.1. Study Design and Study Population

We performed an observational, retrospective study at the National Institute for Infectious Disease “Professor Dr. Matei Balș” (INBI) in Bucharest, Romania, from 1 January 2020 to 31 December 2023. The hospital setting is a tertiary hospital, one of the largest infectious disease hospitals in Bucharest, and a reference center for international public health alerts. During the period when COVID-19 was a Public Health Emergency of International Concern (PHEIC), the hospital was a frontline sanitary unit for COVID-19 confirmed cases during all pandemic waves [26].

All patients included in the study were cases of CDI detected according to the national methodology elaborated by NCSCCD in Romania, based on the European surveillance protocol for CDI, version 2.4 [19,21]. All cases were admitted in the hospital setting during the study period. The cases presented with diarrheal stools or toxic megacolon, pseudomembranous colitis revealed by lower gastro-intestinal endoscopy, or colonic histopathology characteristic of CDI (with or without diarrhea), according to a specimen obtained during endoscopy, colectomy, or autopsy. The etiology was confirmed using the chromatographic immunoassay CerTest *C. difficile* Toxin A+B (Biotec S.L., Zaragoza, Spain), the Xpert *C. difficile* PCR assay (Cepheid, Sunnyvale, CA, USA), or the Biofire Film PCR Array (bioMérieux, Salt Lake City, UT, USA).

A CDI recurrent case was defined as the reemergence of symptoms and subsequent positive laboratory tests between two and eight weeks since the last positive specimen and after treatment completion. A new episode in the same patient was defined as the reemergence of symptoms and subsequent positive laboratory tests eight weeks after the first infection. The CDI cases were defined as complicated if they met one of the following criteria: intensive care unit (ICU) admission, toxic megacolon and colectomy, no symptom improvement following the treatment, recurrences of the infection, or death within 30 days of the initial diagnosis.

Surveillance of CDI cases was passive between 2020 and 2022; the clinicians reported the cases to the Infection Prevention Department (IPC) according to the national methodology. In 2023, an active tool was added to improve the quality of the CDI surveillance data. All medical diagnoses in the hospital informatic system were screened by the IPC team weekly for the keywords (“diff”, “c.diff”, “clostridium”, and “clostridoides”). The electronic patient files that contained the keywords were further reviewed to determine if they met the case definition criteria. All suspected or confirmed cases were reported to the local public health department in a specific surveillance file, which contained data regarding the symptom onset, risk factors, and the classification of the case according to the origin of the infection [21]. CDIs were categorized as healthcare-acquired (HA-CDI), community-acquired (CA-CDI), or unknown origin (UO). HA-CDI was also classified by the origin of the infection being from the hospital setting, a different hospital, long-term care facilities, outpatient care settings, or an unknown medical facility, based on the symptom onset. The case was classified according to the origin of the infection, confirmed by the epidemiologist from the Infection Prevention Department (IPC) after the validation of the data from the surveillance form, according to the surveillance methodology [21].

We excluded outpatients and patients examined in the emergency unit from the analysis. The analysis of antibiotic consumption excluded patients lacking data on prior antibiotic use preceding symptom onset and data from CDI relapse episodes.

### 2.2. Statistical Analysis

We performed a descriptive analysis using demographic and epidemiological data related to confirmed CDI cases, such as the frequency of cases, age, gender, residence, and risk factors, with an emphasis on antibiotic use. Continuous and categorical variables were presented as medians, means, interquartile ranges (IQRs), numbers, and percentages.

The epidemiological indicators analyzed in the study were the number of confirmed cases per year, the annual incidence of CDIs in the hospital setting (number of cases per 10,000 discharged patients), the number of deaths per year, and the case fatality rate (number of deceased persons after the onset of the symptoms or after diagnosis, per 100 confirmed CDI cases) [27]. We also performed a comparative analysis of HAI-CDI and CI-CDI regarding the demographic characteristics and the severity of the cases using χ^2^, Mann–Whitney U, and Kruskal–Wallis H tests. For all tests, a *p*-value of less than 0.05 was considered to be statistically significant. Data collection, analysis, and visualization were performed using Microsoft Excel 365, Version 2108 and Minitab Statistical Software Student Version 14, LLC. (State College, PA, USA), 2021.

### 2.3. Ethical Considerations

The Institutional Bioethics Committee approved the study protocol, with the registration number C11049/28.10.2024. Standard informed consent was required from all participants. Access to data was limited to the IPC team and study investigators, ensuring confidentiality.

## 3. Results

### 3.1. Descriptive Analysis

The study included 618 cases of CDIs reported over a period of 4 years. The median number of CDI cases reported per year was 97, with an IQR of 255 (56–311). More than half of the cases (377, 61%) were reported in 2023, followed by 2022 (112, 18.1%), 2020 (82, 13.3%), and 2021 (47, 7.6%). The number of CDI cases reported monthly increased significantly during 2023 compared to the previous 3-year period (*p* < 0.001, 95% CI [20; 29.4]).

The majority of reported CDI cases (455, 73.6%) were diagnosed as HA-CDI, with a median of 75.5 and an IQR of 167 (49–216). The proportion of HA-CDI cases ranged between 69.2% and 91.5% and varied significantly by year (*X*^2^(3, *N* = 4) = 15.1, *p* = 0.002). A total of 145 (23.5%) cases were CA-CDIs, with a median of 18.5 cases and an IQR of 78 (6–84). The proportion of CA-CDI varied between 8.5% and 27.6% and differed significantly by year (*X*^2^(3, *N* = 4) = 13.1, *p* = 0.005). The median number of CDI cases with an unknown origin (UO-CDI) was three, with an IQR of nine (one–ten). No significant difference was detected.

The annual incidence of CDIs varied from 65.1 cases per 10,000 discharges in 2020 to 211.7 cases per 10,000 discharges in 2023, with a continuously ascending trend.

Table 1 presents the distribution of cases and their annual incidence rate.

The median rate of HA-CDI cases per 10,000 discharged patients was 66.6, with an IQR of 71 (57–128). The rate of HA-CDIs per 10,000 discharges ascended during the study period.

**Figure 1:**
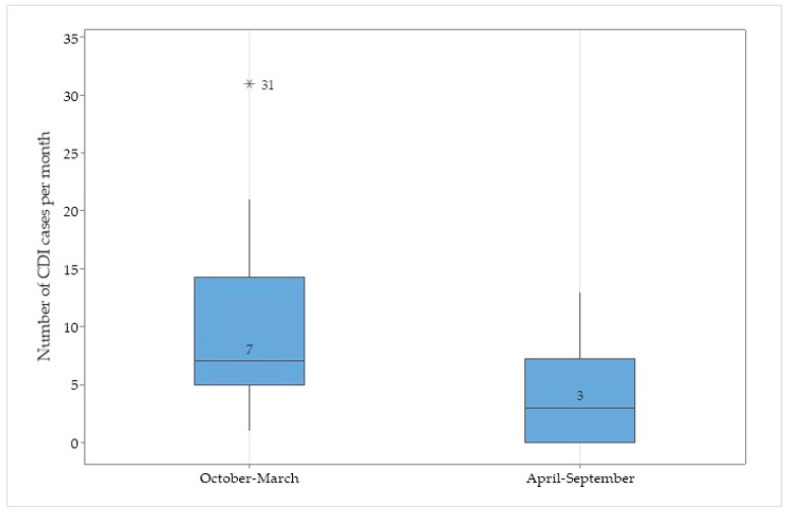
Monthly number of CDI cases reported by season during 2020–2022. (Note: the whiskers indicate the 95th percentiles and the asterisk indicates an outlier).

The median number of CDI cases reported monthly during the 48-month period was 7, with an IQR of 18 (3–21). The Kruskal–Wallis H test showed that there was a statistically significant difference in the number of cases reported monthly between the cold season (October to March) and the warm season (April to September) (*X*^2^(17, *N* = 18) = 6.6, *p* = 0.01) during the first three years of the study, the graphical representation of which is shown in Figure 1. When the fourth year was included in the analysis, no significant differences were found between the monthly number of reported cases (*X*^2^(11, *N* = 12) = 3, *p* = 1), quarterly reported cases (*X*^2^(3, *N* = 4) = 1.5, *p* = 0.7), and half-yearly reported cases (*X*^2^(23, *N* = 24) = 2.8, *p* = 0.9).

Infections were predominantly recorded in females (324, 52.4%) and individuals over 60 years old (383, 63.6%), with the median age of the study population of 68 years old and IQR of 33 (45–78). Most of the cases (489, 79.1%) had urban residences. The demographic characteristics are detailed in Table 2.

From the total number of 455 HA-CDI cases, 232 (51%) were females and 223 (49%) males, resulting in a female-to-male ratio of 1.05 to 1. The median age was 69 with an IQR of 26 (52–78).

The 145 cases of CA-CDI included 86 (59.3%) females and 59 (40.7%) males, resulting in a female-to-male ratio of 1.5 to 1. The median age for this group was 59 years, with an IQR of 44 (31–75).

Most of the cases (464, 75.1%) were diagnosed in the hospital setting. A small percentage of cases were transferred from other hospitals (100, 16.2%) and long-term care facilities (54, 8.7%).

### 3.2. Diagnosis and Clinical Outcomes

Digestive symptoms were reported at admission in 466 cases (75.4%). Overall, 471 (76.2%) cases were diagnosed using chromatographic immunoassays and 71 (11.5%) cases were diagnosed using PCR tests, while, in 76 (12.3%) cases, both methods were used.

The clinical progression of CDI was favorable in 544 cases (88%). However, 75 (12.1%) patients developed severe disease manifestations, with 24 (3.9%) requiring intensive care support. During the four-year period, 53 (8.6%) patients died. The distribution of cases per origin and discharge status is described in Table 3.

For the entire study population, the median length of stay in the hospital was 11 days, with an IQR of 9 days (8–17). The median length of stay for CA-CDI cases was 8 with an IQR of 6 (6–12). The median length of stay for HA-CDI cases was 13 with an IQR of 11 (9–20). The median hospitalization period for CA-CDI cases was significantly shorter than HA-CDI (U = 17,706.5, *p* < 0.001).

Patients requiring intensive care had the longest hospital stay, with a median of 20 days and an IQR of 25 (9–34). The median hospitalization period for deceased patients was 18 days, with an IQR of 18 (8–26).

CDI recurrence was noted in 80 (12.9%) of the evaluated cases. Among the recurrent cases were 45 females and 35 males, with a female-to-male ratio of 1.3 to 1. The median age of these cases was 73 years, with an IQR of 19 (62–81). Fifty-seven recurrent cases were among elderly persons (>65 years). Most of the CDI recurrent cases (72 out of 80 cases) presented with a history of hospitalization. Seven cases were institutionalized, but the records did not mention hospitalization or institutionalization in six cases.

A total of 53 deaths were recorded, comprising 26 females and 27 males, with a median age of 76 years and an IQR of 17 (66–83). Most of the deaths registered were HA-CDI (49 out of 53), while four occurred in patients with CA-CDI. The annual distribution of deaths is shown in Table 4. All deaths occurred exclusively among adults, with four cases registered in the 18–45-year age group, nine cases in individuals aged 46–65 years, and forty in the >65 years age group. Out of the total of 53 deaths registered, the attending physicians documented a link between death and CDI in 18 cases, leading to an overall 2.9% fatality rate in our study population.

No cases of toxic megacolon or fulminant colitis were reported.

### 3.3. Antibiotic Exposure

The antibiotic consumption analysis included data from 390 first CDIs in unique patients. We excluded 80 recurrent CDIs, 92 cases without antibiotherapy, and 56 cases for which data were not available from the analysis. Among the 390 cases analyzed, 153 (39.2%) received single-drug antimicrobial therapy, while 226 (57.9%) received combination therapy. Data regarding the mode of antibiotics administration for 11 patients (2.8%) were unknown.

Half of the CDI cases (196, 50.4%) received third-generation cephalosporins in the three months before the onset of the CDI, while 110 (28.3%) received a beta-lactams and beta-lactam inhibitor (IBL) combination and 80 (20.6%) of cases received carbapenems. The detailed data are presented in Table 5.

**Figure 2:**
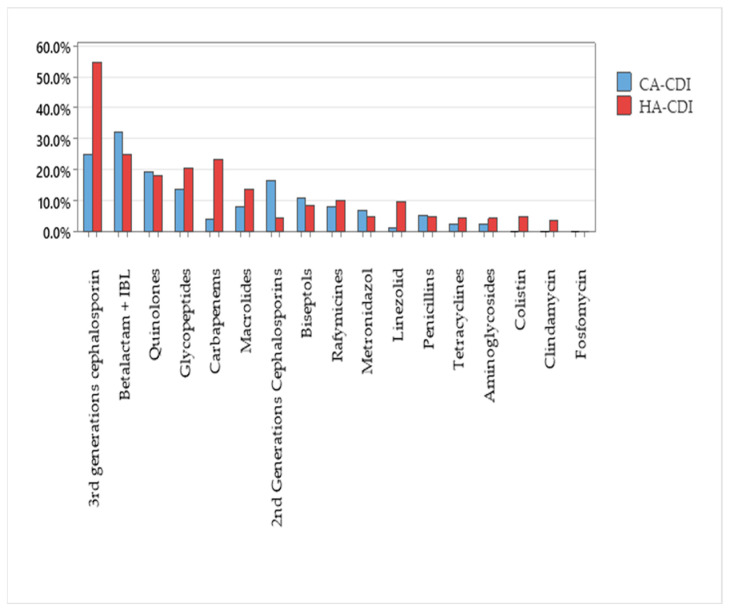
Antibiotic exposure by antibiotic class and origin of CDI case during 2020–2023 (*n* = 390).

The majority of cases included in the antibiotic consumption analysis (315, 80.8%) were HA-CDI, while 72 (18.4%) were CA-CDI and 3 (0.8%) were UA-CDI. Antibiotic exposure, according to the origin of each case, is presented in Figure 2.

### 3.4. Other Risk Factors for CDI

Other risk factors for CDI are presented in Table 6 and include previous treatment with gastric antisecretory agents, immunosuppression, contact with a confirmed CDI case (epidemiological link), and gastrointestinal surgery. Most CDI cases (409, 66.2%) were linked to a previous stay in a hospital.

Additionally, 34 patients (38.2%) had previously used antisecretory agents, and 42 (28.9%) were immunocompromised. Three (2.1%) patients were linked to a CDI case, but 320 (51.8%) had an unknown history of contact with a CDI case.

In HA-CDI, previous treatment with antisecretory agents was registered in 199 (42%) cases, immunosuppression in 165 (36.3%) cases, and direct contact with a CDI case in 12 (2.6%). Almost two thirds of HA-CDI cases (296, 65%) were linked to a previous stay in a hospital, while 42 cases (9.2%) were linked to long-term care facilities. Approximately a quarter of cases included in the study (117, 25.7%) were HA-CDI cases that originated in our hospital, resulting in an overall incidence rate of 23.9 per 10,000 patients discharged. The incidence range varied from 12.8 to 46.5 during the four-year study period.

In CA-CDI cases, 34 (23.4%) had previously used antisecretory agents, 42 (28.9%) were immunocompromised, and 3 (2.1%) were linked to a CDI case. Thirty-seven cases (25.5%) reported hospital admissions in the past year, with 22 (15.1%) of these admissions having occurred within the last three months.

## 4. Discussion

The surveillance of public health provides valuable results that can be used in interventions. The advantage of knowing the actual dimensions of health-related problems goes hand in hand with the need for important human and financial resources, especially during public health emergencies. Continuous surveillance, regardless of other epidemiological challenges, is an important asset of any efficient public health system.

This study, based on data collected through four years of surveillance, highlights the importance of this tool in monitoring the trend of the frequency of CDIs and the main risk factors involved in the development of clinical symptomatic CDIs in hospitalized patients.

Our study results provide insights into CDI in a COVID-19 front-line hospital, and comparisons with current studies and past results may be valuable in identifying new trends and research topics.

### 4.1. Discussion of Descriptive Analysis

The results regarding the annually reported cases in the surveillance system and the calculated incidence of CDI have varied in different studies and reports. We compared our results to European and national data, as the case definition was similar. European annual surveillance reports mention that, in infectious disease hospitals, the CDI incidence is higher than in general or specialized units [20,28].

A Romanian study of nine hospitals, the current unit included, showed that the overall prevalence of CDIs was above 20 per 10,000 patient days, which was much higher than the mean European prevalence [23].

A study from Portugal reported an incidence above 18 cases per 10,000 admitted patients between 2018 and 2022, with a peak of 28.5 cases per 10,000 admitted patients [29].

The influence of the COVID-19 pandemic on the incidence of hospitalized CDI cases was reported in other studies performed in 2020–2022, highlighting the decrease in incidence and the higher severity of these cases, which were explained by the prevention measures implemented at the hospital level [30]. Our study analyzes a long period and points out a very large number of the cases reported in 2023. These findings may have been due to a new active surveillance tool implemented at the hospital level, and underline the importance of the continuous, accurate surveillance and analysis of CDI data. The patients’ medical charts were supplementarily reviewed by the IPC team, and clinicians were consequently trained on the CDI surveillance methodology.

In all four years of the study, HA-CDI was most frequently registered, with its lowest incidence rate found in 2023. A previous study conducted at the study site found that the incidence of HA-CDI did not change between the pre-pandemic (2017–2018) and pandemic (2020–2021) periods, with the incidence of HA-CDI being 56 vs. 61 per 10,000 discharges [31]. The incidence of HA-CDI during the post-pandemic period described in our study almost doubled (116.5 per 10.000 discharges), possibly due to the implementation of an active surveillance system in 2023.

The median age of patients included in our study was lower than the findings reported by other studies, but the most predominant age bracket of our cases was over 60 years old [30,31,32,33]. In order to assess the age-related risk of developing CDI based on age, future studies are considered necessary. The female gender is considered a risk factor for CDI in some studies; our study had a gender ratio favorable to females but found no significant differences between the genders [34].

We observed that most cases originated from urban areas, which could be attributed to better access to healthcare services. Additionally, the higher number of cases in urban areas could be due to increased accessibility to antibiotics, as the main risk factor associated with CDIs.

Our results regarding the seasonality of CDIs suggested that there were differences between cold and warm seasons during the first three years, which is concordant with one metanalysis based on 20 studies from North America, Europe, and Oceania [35]. However, when we added the fourth year of surveillance, the seasonal pattern regarding the number of reported cases was not statistically significant. A Romanian study based in the same hospital setting compared the incidence of HA-CDIs during a pre-pandemic period (2017–2018) and the early pandemic years (2020–2021) and registered no statistically significant differences. In the fourth year of surveillance, the Omicron variant of SARS-CoV-2 was dominant, and the hospital saw continuous activity as a frontline hospital [36,37,38]. This rationale opens new study hypotheses regarding the influence of the COVID-19 pandemic on CDI.

### 4.2. Discussion of Diagnosis and Clinical Outcomes

Most cases of our study were diagnosed using chromatographic immunoassays, similar to another study performed in the western part of the country [39].

Our group’s average length of hospital stay was considerably shorter than the average length of stay observed in the previous study (11 days vs. 36 days), which included patients from another tertiary infectious diseases hospital in Romania [39]. Even when comparing the average length of stay during the pandemic period (2020 and 2021), the average length of hospitalization was lower for our hospital setting (16.5 days vs. 36 days). In 2021, the longest hospitalization period for CDI cases was recorded, with a median of 19 days, suggesting an influence of COVID-19 waves on the clinical outcome of CDIs [30].

The relapse rate observed in our study was higher than that of a similar study conducted in another region of Romania in 2020 and 2021 (12.9% vs. 5%, respectively) [39]. This could be explained by the timeframe of the two studies and changes in the trends of CDIs, and it highlights the heterogeneity of the multiannual surveillance data.

In contrast, in a study performed in Germany over a 4-year pre-pandemic period, the relapse rate was higher (40–65%) [34]. In that longitudinal cohort study, CDIs were associated with a high risk of death [34,40,41].

### 4.3. Discussion of Antibiotic Exposure Results

Broad-spectrum antimicrobials were the most frequently identified risk factor for CDI, due to their significant impact on normal intestinal flora. Any antibiotic use can increase susceptibility to CD, but cephalosporins, β-lactams, and carbapenems increase the risk [42]. The classes of antibiotics mentioned were the three most frequently identified as risk factors in our study. In the COVID-19 era, there was lower antibiotic use. Nevertheless, the studies and Romanian reports show a high antibiotic consumption in the entire country [22,23]. Planning effective strategies for the judicious use of antibiotics requires medical education, motivation, and systemic changes. In Romania, guidelines for antibiotic use were developed and are implemented inconsistently through hospital antimicrobial stewardship [43,44]. For outpatients, antibiotics are provided only with a medical prescription at pharmacies, enforced by legislation [45].

The study does not evaluate the ribotypes of CD, but, in Romania, the significant circulation of a highly moxifloxacin-resistant ribotype 027 at a national level has been reported [23].

### 4.4. Discussion of Other Risk Factors for CDI

On the surveillance form, the most important risk factors assessed were hospitalization, immunosuppression, gastric suppressant and proton pump inhibitors, contact with a confirmed case of CDI, and gastrointestinal surgery.

The frequency of immunosuppression as a risk factor is comparable with the findings in the previously published literature, and the use of gastric acid suppressants was consistently higher than our results [29,46].

Most of these risk factors are individual risk factors. Still, contact with symptomatic CD-infected patients can be prevented through hospital isolation procedures and proper hand hygiene, lowering the risk of HA-CDI.

### 4.5. Strengths and Limitations of the Study

The study’s strengths include the high quality of the data collected by active and passive surveillance over an extended period, resulting in a large sample size. The data were collected in a standardized manner, using surveillance forms according to national and European methodology. Every case was validated and classified according to the case definitions.

The study has several limitations. We did not include data regarding the genomic characteristics of CD. Data regarding the recurrence of CDI were collected only at the hospital level, and patient follow-up was not available.

## 5. Conclusions

The incidence of CDIs in hospitalized patients demonstrated an upward trend each year, with most of the registered cases being HA-CDI. CDIs may lead to prolonged hospitalization, complications, or death.

Active surveillance has improved the detection of cases in our hospital and is essential for characterizing risk factors and identifying possible targets for interventions.

Hospitalization and antibiotherapy, predominantly with third-generation cephalosporins, β-lactams with inhibitor combination, and carbapenems, were the most frequent risk factors registered.

Antibiotic stewardship, medical education, and guidelines can address the consumption of unnecessary antibiotics. However, other risk factors, with a lower frequency, still need to be surveyed.

## Figures and Tables

**Table 1:** The number and incidence rate of CDI cases, according to the origin of infection between 2020 and 2023 (*n* = 618). * The incidence rate is calculated per 10,000 discharges.

CDIs	All CDIs *n (%)*	2020	2021	2022	2023
Frequency of the cases					
Total cases	618	82 (13.3)	47 (7.6)	112 (18.1)	377 (61.0)
HA-CDI	455 (73.6)	68 (82.9)	43 (91.5)	83 (74.1)	261 (69.2)
CA-CDI	145 (23.5)	12 (14.6)	4 (8.5)	25 (22.3)	104 (27.6)
UA-CDI	18 (2.9)	2 (2.4)	0 (0)	4 (3.6)	12 (3.2)
Incidence rate *					
Inpatients discharges		12,427	6888	11,732	17,808
HA-CDI incidence		54.7	62.4	70.7	146.6
CA-CDI incidence		9.7	5.8	21.3	58.4
UA-CDI incidence		1.6	0	3.4	6.7
CA/UA-CDI incidence		11.3	5.8	24.7	65.1

**Table 2:** Demographic characteristics of CDI cases during 2020–2023 (*n* = 618).

Characteristics		(*n*)	(%)
Gender	Female	324	52.4
	Male	294	47.6
Residence	Rural	129	20.9
	Urban	489	79.1
Age groups	0–1	3	0.5
(years)	2–9	44	7.1
	10–19	21	3.4
	20–39	70	11.3
	40–59	87	14.1
	60–69	111	18
	70–79	154	24.9
	80–89	104	16.8
	>90	24	3.9

**Table 3:** Patient status at discharge by origin of infection during 2020–2023 (*n* = 618).

Discharge Status	All CDIs	HA-CDI	CA-CDI	UA-CDI
	*n* = 618 (%)	*n* = 455 (%)	*n* = 145 (%)	*n* = 18 (%)
Improved	544 (88.3)	392 (86.2)	135 (93.1)	17 (94.4)
No significant change	21 (3.4)	14 (3.1)	6 (4.2)	1 (5.6)
Deceased	53 (8.6)	49 (10.7)	4 (2.8)	0 (0)

**Table 4:** Duration of hospitalization (days) for overall and complicated cases.

Year	Total CDI (*n*)	Deceased Cases (*n*)	Length of Stay Median (IQR)	Length of Stay ICU Median (IQR)	Length of Stay Deceased Median (IQR)
2020	82	11	14 (10)	23 (5)	18 (11)
2021	47	2	19 (13)	38.5 (3)	32 (10)
2022	112	13	12 (11)	34 (36)	24 (23)
2023	377	27	11 (7)	13 (12)	14 (16)

**Table 5:** Previous antibiotherapy of first CDI cases during 2020–2023 (*n* = 390).

Antibiotic Classes	Total (390 Cases) *n* (%)	2020 (65 Cases) *n* (%)	2021 (41 Cases) *n* (%)	2022 (78 Cases) *n* (%)	2023 (229 Cases) *n* (%)
Third generation cephalosporins	196 (50.4)	37 (64.9)	23 (48.9)	38 (51.4)	92 (42)
β-lactams + IBL	110 (28.3)	5 (8.8)	9 (19.1)	17 (23)	73 (33.3)
Carbapenems	80 (20.6)	12 (21.1)	17 (36.2)	12 (16.2)	36 (16.4)
Glycopeptides	80 (20.6)	6 (10.5)	5 (10.6)	14 (18.9)	51 (23.3)
Quinolones	73 (18.8)	8 (14)	6 (12.8)	13 (17.6)	44 (20.1)
Macrolides	49 (12.6)	20 (35.1)	11 (23.4)	3 (4.1)	15 (6.8)
Aminoglycosides	17 (4.4)	3 (5.3)	2 (4.3)	8 (10.8)	4 (1.8)
Colistin	16 (4.1)	6 (10.5)	1 (2.1)	3 (4.1)	6 (2.7)
Others	207 (53.1)	20 (35.1)	11 (28.2)	48 (64.9)	123 (56.2)

**Table 6:** The risk factors registered in the study population during 2020–2023.

Risk Factor		*n* (%)
Hospitalization in the previous 12 months	Yes	409 (66.2)
	No	183 (29.6)
	Unk.	26 (4.2)
Immunosuppression	Yes	200 (32.4)
	No	372 (60.2)
	Unk.	46 (7.4)
Gastric acid suppressants and proton-pump inhibitors	Yes	236 (38.2)
	No	275 (44.5)
	Unk.	107 (17.3)
Contact with a confirmed CD case	Yes	15 (2.4)
	No	283 (45.8)
	Unk.	320 (51.8)
Gastrointestinal surgery in the previous 2 weeks	Yes	19 (3.1)
	No	588 (95.1)
	Unk.	11 (1.8)

## Data Availability

The data presented in this study are available on request from the corresponding author due to hospital site methodology regarding confidentiality and data security.
